# Young Children with ASD Use Lexical and Referential Information During On-line Sentence Processing

**DOI:** 10.3389/fpsyg.2016.00171

**Published:** 2016-02-19

**Authors:** Edith L. Bavin, Evan Kidd, Luke A. Prendergast, Emma K. Baker

**Affiliations:** ^1^School of Psychology and Public Health, La Trobe University, MelbourneVIC, Australia; ^2^Research School of Psychology and ARC Centre of Excellence for the Dynamics of Language, Australian National University, CanberraACT, Australia; ^3^Department of Mathematics and Statistics, La Trobe University, MelbourneVIC, Australia; ^4^Olga Tennison Autism Research Centre, La Trobe University, MelbourneVIC, Australia

**Keywords:** sentence processing, autism spectrum disorder, high-functioning, verb bias, instrument bias

## Abstract

Research with adults and older children indicates that verb biases are strong influences on listeners’ interpretations when processing sentences, but they can be overruled. In this paper, we ask two questions: (i) are children with Autism Spectrum Disorder (ASD) who are high functioning sensitive to verb biases like their same age typically developing peers?, and (ii) do young children with ASD and young children with typical development (TD) override strong verb biases to consider alternative interpretations of ambiguous sentences? Participants were aged 5–9 years (mean age 6.65 years): children with ASD who were high functioning and children with TD. In task 1, biasing and neutral verbs were included (e.g., *eat* cake versus *move* cake). In task 2, the focus was on whether the prepositional phrase occurring with an instrument biasing verb (e.g., ‘Chop the tree with the axe’) was interpreted as an instrument even if the named item was an implausible instrument (e.g., candle in ‘Cut the cake with the candle’). Overall, the results showed similarities between groups but the ASD group was generally slower. In task 1, both groups looked at the named object faster in the biasing than the non-biasing condition, and in the biasing condition the ASD group looked away from the target more quickly than the TD group. In task 2, both groups identified the target in the prepositional phrase. They were more likely to override the verb instrument bias and consider the alternative (modification) interpretation in the implausible condition (e.g., looking at the picture of a cake with a candle on it’). Our findings indicate that children of age 5 years and above can use context to override verb biases. Additionally, an important component of the sentence processing mechanism is largely intact for young children with ASD who are high functioning. Like children with TD, they draw on verb semantics and plausibility in integrating information. However, they are likely to be slower in processing the language they hear. Based on previous findings of associations between processing speed and cognitive functioning, the implication is that their understanding will be negatively affected, as will their academic outcomes.

## Introduction

Communication difficulties are a core component of Autism Spectrum Disorder (ASD). Although there has been a body of research regarding how children with ASD differ from children with typical development (TD) in terms of their linguistic development (e.g., see Naigles and Chin, 2015 for an overview), research on how young children with ASD process language in real time is still in its infancy. Such information is potentially valuable: communicative difficulties are characteristic of ASD, and studying their on-line processing has the potential to reveal possible sources of these difficulties.

Lexical information plays an important role in syntactic processing and sentence interpretation (Trueswell et al., 2011). In understanding what others say a listener recovers the structures and meaning of the intended message, building up a conceptual representation as more information is processed. The process is continuous and incremental: as more words are processed they provide syntactic and semantic cues to constrain the ongoing analysis. In this paper, we add to the research on language processing in young children with ASD by reporting on two eye-tracking tasks designed to investigate the extent to which young children with ASD are biased by the semantics of the verb in a sentence to make specific predictions or interpretations, as has been reported for children and adults with TD (e.g., Altmann and Kamide, 1999, 2007; Nation et al., 2003; Mani and Huettig, 2012).

Listeners draw on a number of cues in interpreting sentences, including verb semantics. The semantics of a verb constrains what listeners expect, and their eye movements to a visual display reflect this. Using the visual world eye-tracking paradigm, Altmann and Kamide (1999) tested adults with sentences which contained either a biasing verb (e.g., *eat*) or a neutral verb (e.g., *move*). The visual array included a target (e.g., a cake) and distractors (inedible objects). When the sentence contained a biasing verb (e.g., ‘The boy will *eat* the cake’), the participants’ looking patterns indicated that they anticipated the target before it had been named. That is, immediately on hearing *eat*, they looked at the cake. In contrast, following *move*, it took participants longer to look at the cake. Such biasing constraints were also found by Nation et al. (2003), who tested 10 to 11-year-old children with TD and children who had been identified as having comprehension difficulties. The authors reported similar results for both groups. Similarly, in a study with 3 to 10-year-olds, Borovsky et al. (2012) also found a verb biasing effect. Even at age 2 years, children with good expressive vocabulary anticipated the object of a transitive verb when the sentence contained a semantically biasing verb (e.g., *eat* vs. *see* and *stroke* versus *like*; Mani and Huettig, 2012). That is, the ability to predict the upcoming content based on verb semantics appears to develop early, and this development facilitates sentence interpretation.

While verb biases provide a strong constraint on sentence processing, they can be overridden, as illustrated by Trueswell et al. (1999). In this study test sentences such as ‘Put the frog on the napkin in the box’ were presented in two conditions. In the one-frog condition a frog was sitting on the table, and in the two-frog condition one frog was sitting on a napkin and the other was sitting on the table. The semantics of *put* biases listeners to expect a destination to be named (where something is to be put). When two frogs were included, the adults’ eye movements indicated that they were less likely to assume a destination for the phrase ‘on the napkin.’ The *on*-phrase potentially serves as a noun modifier, identifying which of the two frogs was to be moved and this was confirmed when the intended destination ‘in the box’ was named. That is, the adults drew on the contextual information of two frogs present, but the 5-year-old participants did not. Their eye movements showed they were biased by the verb, predicting that the *on*-phrase was the destination. Thus the 5-year-olds failed to integrate the contextual with the linguistic information.

This finding is consistent with that from Snedeker and Trueswell (2004). They tested 5-year-olds using sentences containing *with* prepositional phrases, and manipulated the type of verb used in test sentences. For example, one test sentence was ‘Tickle the pig with the stick.’ While the sentence is ambiguous, the verb *tickle* biases an instrument reading (i.e., *tickle the pig using the fan*). In contrast, a sentence like ‘Choose the pig with the stick’ biases a noun-modifier interpretation (that is, choose the pig that is holding the stick). The results of the study indicated that the verb semantics biased the children’s on-line processing and interpretations. However, referential context did not strongly affect their processing: even when the context was manipulated to include two pigs in the visual display (one with a stick and one without) there was very little evidence that the children interpreted *with the stick* as noun modification to help them select one of the two pigs.

Another study showed the strength of the verb bias with 5-year-olds (Kidd et al., 2011); in this study plausibility rather than context was manipulated. For example, test sentences included ‘Cut the tree with the leaves,’ but *leaves* are not plausible as an instrument of cutting. Adults were also tested with the results showing that they looked at both the implausible instrument (leaves) and the item that indicated a nominal interpretation of the *with*-phrase, that is, a tree with leaves on it. However, the looking patterns of the 5-year-olds indicated they were likely to accept implausible instruments; that is, they had difficulty overriding the strong verb bias for verbs such as *cut* (which predicts an instrument), even in instances where they were presented with an implausible instrument, in favor of a nominal interpretation.

There is relatively little published research investigating the verb bias in sentence processing for individuals on the autism spectrum. Brock et al. (2008) reported a verb bias for adolescents with ASD; the participants anticipated the target object following a biasing verb. For example, as they heard the verb *stroke*, they looked at the picture of a hamster, the one item in the display that could be predicted by the verb. Other research has also shown that 6 to 9-year-olds with ASD who are high functioning can draw on linguistic context to interpret potentially ambiguous information, which indicates an ability to integrate within-sentence information. For example, using an implicit priming paradigm, Hahn et al. (2015) found that the verb in a sentence influenced which meaning of a potentially ambiguous word [e.g., *bat* – bat (animal) versus bat (in sport)] was identified in the course of sentence processing. This was found for both children with ASD and children with TD. However, we know of no previous research with young school age children with ASD designed to identify if (1) the semantics of a verb biases their predictions or interpretations of which object will be named, as has been shown for young children with TD, or (2) whether the instrument bias is weakened when an implausible instrument is named and a nominal modification interpretation is appropriate. If verb biases are found to be different between children with ASD and children with TD, it would suggest that lexical constraints take longer to develop for young children with ASD.

While 31% of individuals with ASD are estimated to be low functioning, that is with IQ scores ≤ 70 (Baio, 2014), others on the spectrum are high functioning, that is, having IQ scores in the normal range. High functioning individuals often have good structural language knowledge, although there may be language impairment (see Loucas et al., 2008; Bishop, 2010). In a previous eye tracking study, we found that 5 to 7-year-old children with ASD who were high functioning, all with language scores from a standardized language assessment in the normal range, spent proportionally less time looking at a named target than children with TD when processing simple sentences and they took longer to fixate on the target (Bavin et al., 2014). In a second eye tracking study (Bavin et al., 2015), 5 to 9-year-old children with ASD who were high functioning took longer to process disambiguating information that allowed identification of a target object when integrating auditory and visual stimuli. That is, speed of processing of language input was found to be slower in children with ASD. Speed of processing is crucial because if an individual is slow in interpreting intended meanings there will be cumulative effects. Speech is fast and continuous; although language input maybe in the form of singe words (e.g., an answer to a question) in typical conversational interactions and classroom settings typical language input includes connected sentences. Thus, unless a listener’s processing speed allows them to keep up, information is missed and miscommunication is likely to occur.

In the current study, two tasks were conducted. The first investigated if there were differences between processing sentences containing biasing verbs and sentences containing non-biasing verbs for children with ASD who were high functioning and a group of children with TD. In the second task, we compared the two groups to identify if they were influenced similarly when a biasing verb was followed by an implausible instrument or a plausible instrument, or if implausible instruments were interpreted as modifiers of the direct object. The possible impact of potential confounding factors was also investigated. In Brock et al.’s (2008) study with adolescents, some of the variance found in the results across both ASD and TD groups was attributed to low language scores; thus we investigated if participants’ language scores from a standardized assessment contributed to the variance. We also considered the impact of age, given that age was found to be an influencing factor in our previous eye tracking study with children with ASD (Bavin et al., 2015). We also included as covariates scores from a standard attention task and a standard memory task as well as IQ scores.

## Materials and Methods

### Participants

The participants, aged 5–9 years, were 47 children with a diagnosis of ASD and 56 children with TD. ASD status was confirmed using the Autism Diagnostic Observation Schedule-Generic (ADOS-G; Lord et al., 2000) administered by a qualified assessor as part of the current study. The children with TD were screened for autism symptoms with the Lifetime Version of the Social Communication Questionnaire (SCQ; Rutter et al., 2003); all of the children with TD scored below the exclusionary cut-off of 11 (Wiggins et al., 2007). Most of the participants were recruited directly through main stream schools in three districts in the Melbourne Metropolitan Region, Australia. Fifteen in the ASD group were recruited through services or organizations associated with ASD, and three in the TD group were recruited through a university-based registry of families interested in participating in research. (See **Table 1** for details about participants and their scores on the standard assessments).

**Table 1:** Means and standard deviations for the dependent variables for the two groups.

	TD			HASD		
		
	*n*	*M*	*SD*	*n*	*M*	*SD*
Age	56 (42 male)	6.62	1.00	47 (40 male)	6.71	1.00
ADOS						
Calibrated Severity Score	–	–	–	44	5.84	2.01
Language						
Core Score	56	103.00	11.55	46	93.80	19.13
Intelligence						
Full Scale IQ (FSIQ)	56	100.21	10.74	43	98.42	12.45
Auditory Attention	56	10.93	2.77	41	10.46	3.51
Memory	56	111.20	14.37	47	108.06	15.80


The study was carried out with approval from the La Trobe University Human Ethics committee and the Ethics Committee of the Victorian State Department of Education and Training. Written consent was obtained from parents and oral consent from the children who participated. Written consent to recruit and test in the schools was obtained from each school principal.

### Materials

#### Autism Diagnostic Observation Schedule-Generic (ADOS-G; Lord et al., 2000)

Module 3 was used with all participants. To obtain the Calibrated Severity Scores (CSS), we used the revised algorithms by Gotham et al. (2009).

#### Language

Our standard language measure was the Clinical Evaluation of Language Fundamentals – Fourth Edition (CELF-4) – Australian (Semel et al., 2003). The Core Language standard scores were used in the analysis. The subtests contributing to this score are: Word Structure, Recalling Sentences, Formulating Sentences, and Concepts and Following Directions.

#### Intelligence

Intelligence was assessed with either the Wechsler Preschool and Primary Scale of Intelligence – Third Edition (WPPSI-III; Wechsler, 2002) or the Wechsler Intelligence Scale for Children – Fourth Edition (WISC-IV; Wechsler, 2003), depending on the age of the child. Vocabulary, Block Design and Information were used from the WISC-IV and Vocabulary, Block Design and Matrix Reasoning from the WPPSI-III. An estimated full scale IQ (FSIQ) was calculated using an algorithm derived by Sattler and Dumont (2004).

#### Attention

Attention was measured using the Attention task from the Developmental Neuropsychological Assessment – Second Edition (NEPSY-II; Korkman et al., 2007). In this task, participants are presented with a page displaying four different colored circles. They listen to a series of words and are asked to touch the red circle whenever they hear the word ‘red.’

#### Memory

The word list recall task from the Working Memory Battery Test for Children (WMBT-C; Pickering and Gathercole, 2001) was included to measure verbal memory.

#### Eye Tracking Task 1

The first eye tracking task included 16 sentences (see Appendix 1 in **Supplementary Data Sheet 1**). Half contained a verb for which the object could be predicted, as in ‘The boy will *eat* the cake.’ For this sentence the four objects displayed on the monitor were: cake, toy, ball, and cup. Eight other sentences were the same as the biasing sentences with the exception that the verb was switched so that it did not bias listeners to one of the four displayed items, e.g., ‘The boy will *move* the cake.’ The four objects displayed were identical for the two versions of each sentence, creating two versions of the task. Children heard an equal number of biasing and non-biasing sentences and heard either the biasing version (*expected* condition) of a sentence or the non-biasing version (*neutral* condition), not both. The four pictures in the display were distributed across the four corners of the Tobii T120 Eye Tracker V 2.2.8 monitor, and the target appeared in different locations across items. The visual display appeared on the monitor one second before the auditory stimuli started.

#### Eye Tracking Task 2

The task was designed to assess if the children were biased by the verb semantics in the test sentences to interpret a *with*-phrase as an instrument even if it named an implausible instrument (as in Kidd et al., 2011), and the extent to which they interpreted the phrase as nominal modification.

Two conditions were included: half the sentences contained a biasing verb and a *with*-phrase that named an expected instrument (expected condition) as in ‘The girl will cut the cake *with the knife.*’ The other half contained a with-phrase that named an *unlikely* instrument, as in ‘The girl will cut the cake *with the candle.*’ We use the term ‘ambiguous condition’ for the sentences with an implausible instrument, although for both conditions the with-phrase was potentially ambiguous between an instrument and modification interpretation. However, the implausibility of an action such as ‘cutting with a candle’ has the potential to influence responses that override the verb bias, resulting in a modification interpretation rather than instrumental. If the children showed an instrument bias in the ambiguous condition they would look at the single candle, but if they looked at the cake with the candle (as opposed to a cake *without* a candle) they were interpreting the with-phrase as nominal modification. The display for this item for both versions, expected and ambiguous, showed a knife, a cake without a candle, a cake with a candle, and a candle alone. (see Appendix 2 in **Supplementary Data Sheet 1** for a list of items included).

The sentences for tasks 1 and 2 were mixed to form four versions. The children heard either the expected version or the ambiguous version of a sentence, not both, thus creating two versions and each of these versions were reversed to create the other two versions. Children were semi-randomly assigned to one of the four versions. As in task 1, the four pictures in the display were distributed across the four corners of the Tobii T120 Eye Tracker V 2.2.8 monitor, and the target appeared in different locations across items.

### Analyses

#### Task 1

Data were analyzed in 200 ms time blocks. For each child, intervals in which the child looked away for more than 67% of the time were excluded (as in Thothathiri and Snedeker, 2008a,b) because they cannot be considered to reflect processing of visual information. The percentage of omitted trials was 6.32 for the TD group and 9.23 for the ASD group. The proportion of looking time to each item within each interval was converted to an ordered factor variable as 0 (<20%), 1 (≥20% and <80%) and 2 (≥80%), as per Bavin et al. (2014, 2015).

R version 2.13.1 (R Core Team, 2013) was used for the analysis and R package ‘geepack’ (Højsgaard et al., 2006) was adopted. It allowed for the data to be analyzed using Generalized Estimating Equations (GEEs; for example, Hardin, 2005), accounting for the repeated measures and assuming an ordered factor response. An exchangeable error correlation structure was assumed to account for dependency between tasks for each child. In the model we included sentence type (i.e., biased (expected) vs. neutral), diagnostic group and the possible confounds. A second analysis for each task used time to fixate to target instead of mean looking time. A child was deemed to have fixated on Item X by Interval Y if they looked at Item X ≥ 80% of the time either within Interval Y or in any preceding intervals. The response was binary and again modeled using GEEs to compare sentence type.

#### Task 2

Additional to the analysis used for task 1, a further analysis was carried out, a time-dependent GEE analysis where the time interval was included as a factor, allowing for non-linear change over time. This allowed us to analyze differences in proportion of looking between time intervals. We also assessed whether children were more likely to look at the named instrument (e.g., the candle) than at the item that represented a nominal modification interpretation (e.g., the cake with the candle) within a specific interval. For this analysis, a difference score was calculated for each item and child by taking the difference between fixation responses for the instrument and modification interpretations and then averaging these across items for each child. A Wilcoxon signed rank test was then used to test whether the average difference score was significantly different than zero.

## Results

### Task 1

**FIGURE 1:**
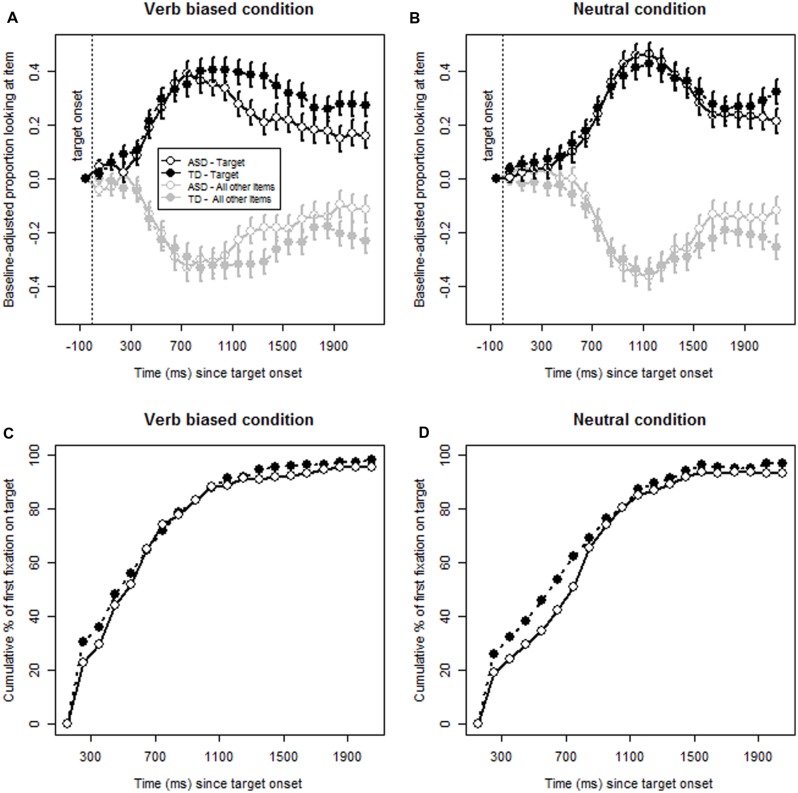
**Task 1: **(A,B)** Proportion of looking to the items from target onset in the verb biased and neutral conditions.** **(C,D)** Fixation patterns from target onset in the verb biased and neutral conditions.

Inspection of the data from task 1 suggested children were already looking at the target more than expected by chance prior to its onset. Thus, preliminary to the first analyses, we plotted children’s looking time using a difference score — mean proportions of looking adjusted for the base-line proportion of looking, that is the proportion of looking at the target in the period 200 ms prior to target onset (–200 to 0 ms). **Figure 1A** illustrates the looking patterns by group (TD and ASD) for the adjusted mean proportion of looking at target (plotted per 100 ms) versus the other three items displayed on the monitor in the biasing verb condition. **Figure 1B** shows the equivalent for the neutral condition.

#### Analysis 1: Proportion of Looking

The initial analysis showed a significant difference across conditions (biasing vs. neutral). Overall, children looked at the target more in the verb biased condition in the intervals 200–400 ms, 400–600 ms, and 600–800 ms. The respective odd ratios (OR), Confidence intervals (CI) and *p*-values were: (*OR* = 1.311, *CI* = [0.985, 1.743] *p* = 0.06; *OR* = 2.071, *CI* = [1.587, 2.703] *p* < 0.001; *OR* = 1.995, *CI* = [1.506, 2.642] *p* = 0.001). Thus we examined the results for each condition separately.

For the *biasing condition* a trend for Diagnosis was not evident until the 1000–1200 ms interval post target onset and this was significant for the subsequent intervals of the analysis time period, that is, through the 1800–2000 ms interval post target onset. **Table 2** summarizes the results of the analysis for the biasing verb sentences for each 200 ms interval from the 600–800 ms interval. (Results for the earlier intervals are shown in **Supplementary Table S1**). The groups differed in that the ASD group looked away from the target to the other pictures more in this time period than did the TD group.

**Table 2:** Task 1: Verb biased condition.

	600–800	800–1000	1000–1200	1200–1400	1400–1600	1600–1800	1800–2000
	
	*OR*	*OR*	*OR*	*OR*	*OR*	*OR*	*OR*
	95% *CI*	95% *CI*	95% *CI*	95% *CI*	95% *CI*	95% *CI*	95% *CI*
	*p*	*p*	*p*	*p*	*p*	*p*	*p*
Diagnosis	0.77	1.05	1.49	2.41	1.88	1.93	1.70
	0.51–1.16	0.68–1.60	0.90–2.45	1.46–3.96	1.15–3.08	1.18–3.14	1.03–2.80
	0.209	0.828	0.118	0.001	0.012	0.009	0.038
Language	0.99	1.01	1.01	1.01	0.99	0.99	1.01
	0.97–1.01	0.99–1.03	0.99–1.03	0.99–1.03	0.97–1.01	0.97–1.02	0.99–1.04
	0.305	0.342	0.256	0.474	0.373	0.601	0.266
Age	1.09	0.88	0.87	1.09	1.11	1.35	1.53
	0.89–1.35	0.73–1.05	0.70–1.09	0.83–1.43	0.84–1.47	1.04–1.75	1.20–1.94
	0.402	0.162	0.224	0.544	0.467	0.025	0.001
FSIQ	1.00	1.02	1.02	1.02	1.01	0.99	0.98
	0.98–1.03	0.99–1.04	1.00–1.05	0.99–1.05	0.98–1.04	0.96–1.03	0.95–1.01
	0.866	0.159	0.073	0.141	0.363	0.697	0.144
Attention	0.96	0.90	0.90	0.94	0.97	0.99	0.96
	0.88–1.04	0.83–0.99	0.81–0.99	0.86–1.04	0.88–1.06	0.91–1.07	0.88–1.05
	0.330	0.026	0.037	0.224	0.476	0.777	0.368
Memory	1.01	1.00	1.00	0.99	1.00	1.01	1.00
	0.99–1.02	0.99–1.01	0.98–1.01	0.98–1.01	0.98–1.02	0.99–1.03	0.99–1.02
	0.365	0.975	0.662	0.415	0.910	0.509	0.560


None of the covariates had a significant impact on the results prior to 800–1000 ms post target onset. As shown in **Table 2**, Attention contributed significant variance in the 800–1000 ms and 1000–1200 ms intervals; children with higher attention scores, regardless of Diagnosis, looked proportionally less at the target in these intervals. The only other covariate to contribute significantly was Age; this was at the end of the analysis window in the last two intervals: 1600–1800 ms and 1800–2000 ms. The older children, regardless of Diagnosis, looked proportionally less in those time intervals; that is, they looked away faster from the target than the younger children.

In the *neutral condition*, Diagnosis was significant following the target onset through the 200–400 ms interval, with the TD group looking more at the target: 2.75, [1.65–4.58] *p <* 0.001 and 1.71, [1.09–2.66] *p* = 0.019. In addition, as can be seen in **Table 3**, there was a trend for Diagnosis from the 1400–1600 ms interval with a significant group difference in the following interval (1600–1800 ms). The ASD group looked less at the target in these intervals. None of the covariates added significant variance. (**Supplementary Table S2** shows the results for the intervals prior to 600–800 ms post target onset).

**Table 3:** Task 1: Neutral condition.

	600–800	800–1000	1000–1200	1200–1400	1400–1600	1600–1800	1800–2000
	
	*OR*	*OR*	*OR*	*OR*	*OR*	*OR*	*OR*
	95% *CI*	95% *CI*	95% *CI*	95% *CI*	95% *CI*	95% *CI*	95% *CI*
	*p*	*p*	*p*	*p*	*p*	*p*	*p*
Diagnosis	1.21	1.10	1.15	1.35	1.53	1.78	1.58
	0.81–1.82	0.68–1.80	0.74–1.78	0.80–2.29	0.94–2.49	1.09–2.90	0.92–2.73
	0.353	0.687	0.542	0.262	0.088	0.021	0.099
Language	1.00	0.99	0.99	1.01	1.01	1.00	1.01
	0.98–1.02	0.97–1.01	0.97–1.01	0.98–1.03	0.99–1.03	0.98–1.02	0.99–1.04
	0.788	0.442	0.390	0.550	0.444	0.978	0.355
Age	1.06	1.01	0.96	1.08	1.09	1.17	1.22
	0.84–1.34	0.80–1.27	0.77–1.21	0.82–1.42	0.85–1.40	0.90–1.51	0.92–1.62
	0.605	0.944	0.749	0.588	0.493	0.243	0.170
FSIQ	1.01	1.00	1.01	1.00	1.00	1.00	0.99
	0.98–1.03	0.98–1.03	0.99–1.03	0.97–1.03	0.97–1.03	0.97–1.04	0.96–1.02
	0.628	0.719	0.353	0.935	0.995	0.778	0.406
Attention	0.94	1.00	1.01	1.02	0.99	0.97	0.92
	0.88–1.01	0.93–1.07	0.94–1.09	0.94–1.11	0.92–1.07	0.89–1.05	0.84–1.02
	0.075	0.900	0.745	0.609	0.796	0.459	0.106
Memory	1.00	1.00	1.00	0.99	0.99	0.99	1.00
	0.98–1.01	0.99–1.02	0.98–1.02	0.97–1.01	0.98–1.01	0.98–1.01	0.98–1.01
	0.509	0.867	0.841	0.492	0.352	0.408	0.708


#### Analysis 2: Fixation Analysis

**Figures 1C,D** show the results of the fixation analysis; similarities between the groups can be seen. In the Neutral condition, a trend for Diagnosis began in the 200–400 ms interval (1.499, [0.936, 2.398] *p* = 0.092) with significant group differences in the 400–600 ms interval (1.527, [1.017, 2.293] *p* = 0.041), and the 600–800 ms interval (1.539, [1.055, 2.244] *p* = 0.025). In these time intervals, more children in the TD group fixated on the target.

### Task 2

#### Proportion of Looking

**FIGURE 2:**
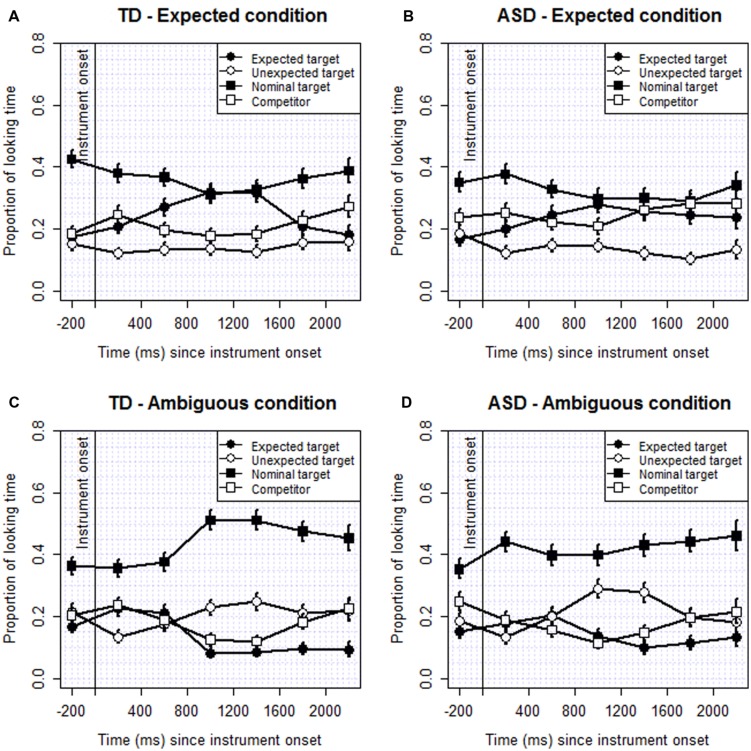
**Task 2: (A–D) Proportion of looking to the four items by group, expected and ambiguous conditions**.

**Figure 2** illustrates the looking patterns by group for the two sentence types, expected and ambiguous. **Tables 4**–**6** show the results of the analyses of these data. They include the odds ratio, 95% CI and *p-*values for the proportion of looking time to the ET, e.g., knife (**Table 4**), UT, e.g., candle (??), and the *complex nominal*, e.g., cake with the candle; that is, proportion of looking at the nominal that could be interpreted as modified by the with-phrase (??). The results are presented from the 400–600 ms time interval post target onset (See **Supplementary Tables S3**–**S5** for results of the earlier time intervals).

**Table 4:** Task 2: Expected target.

	400–800			800–1200			1200–1600			1600–2000			2000–2400		
					
	*OR*	*CI*	*p*	*OR*	*CI*	*p*	*OR*	*CI*	*p*	*OR*	*CI*	*p*	*OR*	*CI*	*p*
Type	1.89	1.21–2.95	0.005	8.64	3.89–19.21	<0.001	5.23	2.78–9.84	<0.001	2.60	1.45–4.67	0.001	2.51	1.35–4.67	0.004
Diagnosis	1.21	0.72–2.06	0.474	1.03	0.59–1.82	0.905	1.32	0.72–2.41	0.372	0.73	0.43–1.25	0.250	0.81	0.48–1.35	0.418
Language	0.98	0.96–1.00	0.082	1.01	0.97–1.04	0.720	1.00	0.97–1.02	0.906	0.99	0.97–1.02	0.622	0.98	0.96–1.01	0.177
Age	0.83	0.63–1.08	0.170	1.07	0.84–1.36	0.576	1.00	0.81–1.25	0.964	1.07	0.82–1.4	0.597	0.97	0.76–1.24	0.794
FSIQ	1.00	0.98–1.03	0.724	0.99	0.96–1.02	0.642	0.99	0.96–1.01	0.358	0.99	0.96–1.02	0.508	1.03	1.00–1.06	0.107
Attention	0.96	0.88–1.06	0.436	0.96	0.85–1.08	0.479	0.97	0.89–1.05	0.470	0.99	0.91–1.08	0.822	0.92	0.84–1.00	0.050
Memory	1.00	0.98–1.02	0.843	1.00	0.98–1.02	0.833	1.01	0.99–1.03	0.307	1.00	0.98–1.03	0.702	1.00	0.98–1.02	0.853


**Table 5:** Task 2: Unexpected target.

	**400–800**			**800–1200**			**1200–1600**			**1600–2000**			**2000–2400**		
					
	***OR***	***CI***	***p***	***OR***	***CI***	***p***	***OR***	***CI***	***p***	***OR***	***CI***	***p***	***OR***	***CI***	***p***
Type	0.58	0.31–1.10	0.098	0.48	0.27–0.86	0.013	0.41	0.24–0.68	0.001	0.62	0.36–1.06	0.080	0.70	0.41–1.20	0.193
Diagnosis	0.89	0.45–1.75	0.738	0.80	0.79–1.31	0.380	1.01	0.63–1.60	0.977	1.52	0.88–2.62	0.135	1.03	0.63–1.68	0.898
Language	1.02	0.98–1.06	0.380	1.00	0.97–1.03	0.996	1.00	0.97–1.02	0.732	1.00	0.98–1.03	0.928	1.00	0.98–1.03	0.726
Age	1.09	0.74–1.61	0.657	0.86	0.68–1.10	0.239	1.07	0.84–1.38	0.571	1.20	0.90–1.59	0.218	1.03	0.80–1.31	0.835
FSIQ	1.01	0.97–1.06	0.612	1.02	0.99–1.05	0.181	1.01	0.98–1.04	0.376	1.00	0.97–1.03	0.792	0.99	0.96–1.02	0.443
Attention	1.07	0.94–1.21	0.332	1.08	1.00–1.18	0.059	1.00	0.89–1.11	0.947	1.08	0.95–1.22	0.236	1.09	0.99–1.21	0.090
Memory	0.99	0.97–1.01	0.332	0.99	0.98–1.01	0.577	1.00	0.98–1.02	0.706	1.01	0.98–1.03	0.535	1.02	1.00–1.04	0.029


**Table 6:** Task 2: Nominal target.

	**400–800**			**800–1200**			**1200–1600**			**1600–2000**			**2000–2400**		
					
	***OR***	***CI***	***p***	***OR***	***CI***	***p***	***OR***	***CI***	***p***	***OR***	***CI***	***p***	***OR***	***CI***	***p***
Type	0.81	0.58–1.12	0.195	0.39	0.26–0.60	<0.001	0.42	0.29–0.62	<0.001	0.48	0.33–0.69	<0.001	0.64	0.46–0.90	0.011
Diagnosis	1.24	0.87–1.77	0.239	1.34	0.97–1.86	0.080	1.35	0.95–1.91	0.092	1.27	0.82–1.96	0.291	0.92	0.59–1.45	0.736
Language	1.00	0.99–1.02	0.616	1.00	0.99–1.02	0.616	1.00	0.98–1.01	0.669	1.00	0.98–1.02	0.865	1.00	0.98–1.02	0.953
Age	0.97	0.82–1.15	0.734	0.88	0.77–1.01	0.071	0.89	0.76–1.05	0.181	0.94	0.74–1.18	0.569	0.97	0.78–1.20	0.766
FSIQ	0.97	0.95–0.99	0.003	0.98	0.96–0.99	0.005	1.00	0.98–1.02	0.908	1.01	0.99–1.04	0.331	1.01	0.99–1.04	0.269
Attention	1.02	0.96–1.09	0.514	1.03	0.97–1.08	0.316	1.03	0.97–1.09	0.386	1.01	0.94–1.09	0.778	0.97	0.90–1.04	0.38
Memory	1.01	1.00–1.03	0.059	1.01	1.00–1.02	0.178	1.00	0.98–1.01	0.577	0.98	0.96–1.00	0.008	0.98	0.97–1.00	0.083


For the proportion of looking to the ET there was a significant effect of sentence type from the 400–800 ms interval through the 1600–2400 ms interval post instrument onset. A greater proportion of looking to the ET was shown in the expected condition than in the ambiguous condition. However, there was no significant group effect. For the proportion of looking to the UT there was a significant effect of sentence type for the 800–1200 ms and 1200–1600 ms intervals. The proportion of looking to the UT was higher in the ambiguous condition than the expected condition (i.e., the opposite of the ET analysis). For proportion of looking to the *complex nominal* target (NT) a significant effect of sentence type was evident from the 800–1200 ms interval through the 2000–2400 ms interval, the last interval of the analysis window. A greater proportion of looking to the NT was evident in the ambiguous condition than in the expected condition (see **Figure 2**). Diagnosis did not make a significant contribution in any of the analyses, and nor did Language, Age, or Attention. In the analysis on the proportion of looking to the NT across the two groups IQ contributed some of the variance in the 400–800 ms and 800–1200 ms intervals with higher scores associated with less looking. In one interval for the NT (1600–200 ms) memory had some impact; in this interval those children with higher memory scores had a lower proportion of looking.

The time model analysis showed that, compared to looking in the 0–400 ms interval following instrument onset, in the ambiguous condition both the ASD and TD groups were looking significantly more at the NT by 800–1200 ms post onset (ASD: 1.78, [1.34–2.38]; TD: 2.01, [1.42–2.84]). A significant increase in looking to the NT in the ambiguous condition persisted following this interval. However, in the expected condition significant differences were not detected in any interval for either group; however, there was a slight decrease in looking for the ASD group in the 800–1200 ms interval (0.68, [0.50–0.94]).

In summary, for sentences that included an ET based on the verb semantics, we found a significantly greater proportion of looking to the plausible instrument named in the prepositional phrase (e.g., the knife). In contrast, in the ambiguous condition there was a significantly greater proportion of looking to the implausible target named in the prepositional phrase (e.g., the candle). That is, the children were influenced by the semantics of the verb to interpret the with-phrase as an instrument phrase, and looked at the named instrument in these instances. However, we also found a significantly larger proportion of looking indicating a nominal modification interpretation of the prepositional phrase in the ambiguous condition than in the expected condition, and this was found for both groups. That is, the children looked more, for example, to the tree with leaves on it and the cake with the candle on it in the ambiguous condition. The results suggest that both groups were capable of overriding the verb bias, instead making the more plausible nominal interpretation for the with-prepositional phrase.

#### Fixation Analysis

**FIGURE 3:**
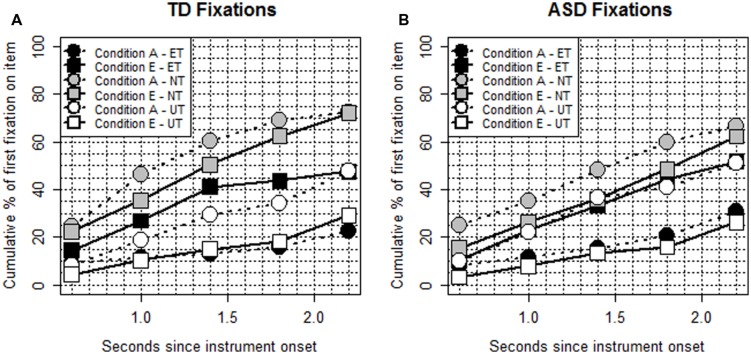
**Task 2: (A,B) Fixation patterns by group from instrument onset**.

**Figure 3** illustrates the fixation patterns for the three types of targets by condition. Fixation on the ET differed significantly between the two conditions, with significantly more children fixating on the ET in the expected condition than in the ambiguous condition. The odds ratios, 95% CIs and *p-*values for the cumulative percentages fixating for the five time intervals from 400 to 2400 ms post target onset were as follows: 1.71, [1.01–2.88] *p* = 0.044; 2.98, [1.94–4.60] *p <* 0.001; 4.17, [2.77–6.28] *p <* 0.001; 3.84, [2.68–5.50] *p <* 0.001; and 2.88, [1.90–4.36] *p <* 0.001. Diagnosis did not have a significant effect on these results.

Fixation on the UT also differed significantly between the conditions, with more fixation on the UT in the ambiguous condition. The odds ratio, 95% CI and *p-*values were as follows: 0.45 [0.22–0.93] *p* = 0.031; 0.48 [0.28–0.82] *p* = 0.007; 0.38 [0.25–0.57] *p* < 0.001; 0.43 [0.29–0.65] *p* < 0.001; and 0.45 [0.30–0.66] *p* < 0.001. That is, fixation on the ET and UT depended on which item the children heard in the with-phrase. Again, Diagnosis was not significant in any of the intervals. When compared to the ambiguous condition, there was evidence of decreased chances of fixation on the NT in the expected condition in the intervals 800–1200 ms and 1200–1600 ms (0.66 [0.43–1.00] *p* = 0.048; 0.64 [0.43–0.94] *p* = 0.024). Additionally, the TD group fixated more to the NT than did the ASD group in intervals 800–1200 ms, 1200–1600 ms, and 1600–2000 ms: 1.59 [1.13–2.24] *p* = 0.007; 1.68 [1.2–2.35] *p* = 0.003; and 1.69 [1.17–2.44] *p* = 0.005, suggesting that the TD group were more sensitive to plausibility information.

Our final analysis on the fixation data compared fixation to the ET vs. NT in the expected condition and fixation to the UT vs. NT in the ambiguous condition by group. In the expected condition, the results showed a significant result for the ET vs. NT for the TD group in the intervals 1600–2000 ms and 2000–2400 ms (*p* = 0.004 and *p* = 0.002) with more children fixated on the NT. However, while the differences were not significant for the ASD group (*p* = 0.901 and *p* = 0.817), as can be seen in **Figure 3**, there was a gradual increase in fixation to the NT toward the end of the analysis window and a drop off in looking to the ET, suggesting that the difference in fixation to the ET and NT for the ASD group would continue to increase. For the NT vs. UT in the ambiguous condition there were significant differences for both groups, with more children fixated on the NT and where there was stronger evidence for this for the TD group (TD: all *p* < 0.008, ASD: all *p* < 0.032).

## Discussion

In summary, for task 1, in the Biasing condition the proportion of looking at target increased for both groups more rapidly than in the Neutral condition. That is, biasing verbs assisted the children to identify the target. However, in the neutral condition, when the verb provided no clues to identify the object, processing was much slower overall, and especially so for the ASD group. For both groups, as shown in **Figure 1B**, the highest mean proportion of looking based on the difference score in the neutral condition was reached at about 1200 ms, whereas it was at about 700–800 ms in the biasing condition. In the biasing condition, once they had identified the target the ASD group looked away significantly more quickly than the TD group. In contrast, in the neutral condition the proportion of looking for the ASD group leveled between1600 and 1800 ms following target onset before dropping, but for the TD group looking to target increased from about 1800 ms.

The fixation analysis showed that more children overall fixated to the target earlier in the biasing condition than in the neutral condition, and in the neutral condition fewer children in the ASD group than the TD group fixated on the target in the 200–800 ms time window. That is, they were more likely to fixate on the target later than children in the TD group.

Why would the ASD group look away in the biasing condition more rapidly than the TD group? In a previous eye tracking study (Bavin et al., 2014) we found that children with ASD looked away from the target more rapidly than children with TD. We suggested that the children with ASD were more interested in the visual material, reflecting a preference for visual information over auditory (Kamio and Toichi, 2000). This possibility also applies to the current results. The children with ASD did match the auditory and visual stimuli; however, they were likely to then view the other items in the visual display.

The study covered a 4 year age range and the results indicate that, regardless of diagnosis, the older children in the sample looked away from the target more quickly than the younger children, suggesting that older children may rapidly use verb semantics to predict upcoming sentence content and require less time to confirm that prediction. Attention also influenced the results in the biasing condition. Overall, children with lower attention scores had a higher proportion of looking to the target, suggesting that more time was required to confirm their choice.

The results from task 1 indicate that if language stimuli contain highly constraining semantic information on crucial sentential constituents, such as a biasing verb, then children with ASD process language similarly to children with TD. This is an important component of the sentence processing mechanism: the ability to anticipate information based on reliable constraints on interpretation (Pickering and Garrod, 2013; Dell and Chang, 2014). The effect for diagnosis in the neutral condition is indicative of a general slowing of processing for the children with ASD in the absence of constraining linguistic information.

The results of task 2 also provided evidence for the verb bias. The verbs included in the test sentences had an instrument bias and participants in both groups looked at the item named in the with-phrase (the expected instrument in the expected condition and the implausible instrument in the ambiguous condition), interpreting the with-phrase as naming an instrument and this was apparent from the 400–800 ms interval post instrument onset. The proportion of looking times to the named target and NT did not differ significantly, however, indicating that both groups were capable of considering alternative syntactic analyses of a sentence in parallel. That the nominal modification interpretation was more likely when the with-phrase identified an implausible target suggests that the children were drawing on and integrating their general knowledge of the world with their knowledge of verb semantics and syntax. Based on our measure of fixation, the TD group were likely to consider alternative interpretations of the with-phrase in both conditions by the end of the analysis period, but for the ASD group alternative interpretations were slower to emerge in the expected condition. The ASD group were quicker to consider the alternative structural analysis, inconsistent with the verb bias, when an implausible instrument was named in the prepositional phrase.

Previous research has shown verb semantics as a reliable cue to interpretation for young children (e.g., Trueswell and Gleitman, 2004). Kidd et al.’s (2011) study with 5-year-olds tested sentences containing ambiguity of instrument and modifier interpretations. The children showed a reliance on verb semantics, that is ‘bottom-up’ lexical cues. However, while the study used eye-tracking as a measure of on-line processing, the task required children to act out the test sentence, thus requiring a behavioral response. It is possible that this feature of the task influenced their interpretation, since children would have been planning motor responses as the sentence unfolded and may have committed to an instrument response from which they could not recover (Meroni and Crain, 2002). In contrast, the current study required no behavioral response, and the children’s eye movements clearly showed that they considered a nominal modification interpretation of the with-phrase as well as an instrumental interpretation. This is the clearest demonstration to date that young children are capable of integrating top-down and bottom-up cues during sentence processing to build multiple potential parses of a sentence.

We found no age effect in task 2 and so assume that the 5-year-olds were as likely to follow lexical (verb) constraints as the older children. This result reveals a verb bias for individuals with ASD who are high functioning at a much younger age than previously shown. As shown in task 1, if a sentence contains a verb that provides no bias, that is, in less predictable contexts, young children with ASD may not process language as rapidly as children with TD. There was some indication of slower processing for the ASD group in task 2 also, in terms of fixating on an alternative structural interpretation when the with-phrase named a plausible instrument.

In previous research, we reported slower processing for children with ASD when they were required to disambiguate information (Bavin et al., 2015). The slower processing shown in both tasks in the current study indicates that for young children with ASD predicable contexts are favored. Predictions made on the basis of reliable cues, which include verb semantics, allows rapid processing of the linguistic input (Pickering and Garrod, 2013). It is significant, therefore, that the children with ASD did make predictions during sentence processing. However, further research is needed in order to explain why their sentence processing is slower compared to children with TD in less predictable contexts. Associations between processing speed and intellectual or cognitive functioning have been reported (Kail and Salthouse, 1994; Miller et al., 2006; Marchman and Fernald, 2008); thus children with ASD may be more vulnerable for poor academic outcomes. We suggest that they might benefit if the sentences used in talking to them were not lengthy, or if they were presented at a slower rate than is typical. This would allow additional time for the children to process the content before additional information is presented.

## Authors Contributions

ELB and EK contributed substantially to the conception of the work. EKB contributed substantially to the collection of data, LP and EKB contributed substantially to the analysis. All authors contributed substantially to interpreting the data, drafting the paper, and revising the drafts for intellectual content. The final version has been read and approved by all authors, and all authors have agreed to be accountable for all aspects of the work.

## Conflict of Interest Statement

The authors declare that the research was conducted in the absence of any commercial or financial relationships that could be construed as a potential conflict of interest.

## Abbreviations

ET: expected target
NT: nominal target
UT: unexpected target
