# Supplementary material for: Young Children with ASD Use Lexical and Referential Information During On-line Sentence Processing
Source: Front Psychol. 2016 Feb 19;7:171. doi: 10.3389/fpsyg.2016.00171 (PMC4759258; doi:10.3389/fpsyg.2016.00171)
Supplement: Supplementary file 1 [file Tables_S.1-S.5.DOCX]

Table S.1

*Task 1 Verb Biased Condition*

|  | -200-0 | 0-200 | 200-400 | 400-600 |
| --- | --- | --- | --- | --- |
|  | OR  95% CI  *p* | OR  95% CI  *p* | OR  95% CI  *p* | OR  95% CI  *p* |
| Diagnosis | 1.18  0.66-2.11  .571 | 0.93  0.57-1.53  .779 | 1.24  0.83-1.86  .297 | 1.01  0.70-1.46  .967 |
| Language | 0.99  0.96-1.02  .474 | 0.99  0.97-1.02  .612 | 0.99  0.97-1.01  .266 | 0.99  0.97-1.00  .095 |
| Age | 1.06  0.79-1.41  .700 | 1.19  0.94-1.51  .155 | 0.96  0.79-.17  .683 | 0.92  0.75-1.13  .414 |
| FSIQ | 1.00  0.97-1.03  .866 | 1.00  0.98-1.03  .877 | 0.99  0.97-1.01  .455 | 0.98  0.96-1.01  .165 |
| Attention | 1.00  0.90-1.11  .988 | 1.04  0.96-1.12  .377 | 1.00  0.93-1.07  .922 | 0.97  0.90-1.05  .473 |
| Memory | 1.00  0.98-1.02  .815 | 0.98  0.97-1.00  .099 | 1.01  0.99-1.02  .271 | 1.01  1.00-1.02  .212 |

Table S.2

*Task 1 Neutral Condition*

|  | -200-0 | 0-200 | 200-400 | 400-600 |
| --- | --- | --- | --- | --- |
|  | OR  95% CI  *p* | OR  95% CI  *p* | OR  95% CI  *p* | OR  95% CI  *p* |
| Diagnosis | 1.26  0.72-2.23  .421 | 2.75  1.65-4.58  <.001 | 1.71  1.09-2.66  .019 | 1.45  0.91-2.31  .119 |
| Language | 0.99  0.97-1.02  .567 | 0.98  0.96-1.00  .079 | 1.00  0.98-1.02  0.83 | 1.01  0.99-1.03  .447 |
| Age | 0.89  0.68-1.18  .429 | 0.92  0.74-1.15  .478 | 1.03  0.81-1.32  .799 | 1.05  0.83-1.34  .674 |
| FSIQ | 1.00  0.97-1.04  .818 | 1.02  0.99-1.04  .169 | 1.00  0.98-1.03  0.924 | 0.99  0.97-1.02  .592 |
| Attention | 1.01  0.90-1.12  .903 | 0.97  0.88-1.08  .602 | 0.96  0.88-1.05  .428 | 0.96  0.89-1.03  .256 |
| Memory | 1.01  0.99-1.02  .424 | 1.01  1.00-1.03  .081 | 1.02  1.00-1.03  .089 | 1.01  0.99-1.02  .311 |

Table S.3

*Task 2 Expected Target*

|  | -400-0 | | | 0-400 | | |
| --- | --- | --- | --- | --- | --- | --- |
|  | OR | CI | *p* | OR | CI | *p* |
| Type | 1.67 | 0.81-3.44 | .167 | 1.27 | 0.76-2.12 | .359 |
| Diagnosis | 1.94 | 0.96-3.91 | .065 | 1.51 | 0.95-2.38 | .081 |
| Language | 1.01 | 0.98-1.03 | .697 | 0.99 | 0.97-1.01 | .521 |
| Age | 1.04 | 0.73-1.48 | .818 | 0.96 | 0.76-1.21 | .729 |
| FSIQ | 0.99 | 0.96-1.03 | .749 | 1.01 | 0.99-1.03 | .415 |
| Attention | 0.90 | 0.81-1.00 | .040 | 0.98 | 0.91-1.06 | .637 |
| Memory | 1.02 | 0.99-1.04 | .140 | 1.00 | 0.98-1.01 | .709 |

Table S.4

*Task 2 Unexpected Target*

|  | -400-0 | | | 0-400 | | |
| --- | --- | --- | --- | --- | --- | --- |
|  | OR | CI | *p* | OR | CI | *p* |
| Type | 0.77 | 0.42-1.42 | .400 | 0.62 | 0.32-1.21 | .160 |
| Diagnosis | 0.80 | 0.45-1.42 | .445 | 1.08 | 0.55-2.11 | .828 |
| Language | 0.99 | 0.96-1.02 | .405 | 1.02 | 0.98-1.05 | .289 |
| Age | 1.03 | 0.79-1.35 | .822 | 0.92 | 0.71-1.20 | .555 |
| FSIQ | 1.00 | 0.96-1.03 | .836 | 1.00 | 0.96-1.03 | .864 |
| Attention | 1.13 | 1.02-1.25 | .019 | 0.96 | 0.86-1.07 | .478 |
| Memory | 0.98 | 0.97-1.00 | .040 | 0.99 | 0.97-1.01 | .417 |

Table S.5

*Task 2 Nominal Target*

|  | -400-0 | | | 0-400 | | |
| --- | --- | --- | --- | --- | --- | --- |
|  | OR | CI | *p* | OR | CI | *p* |
| Type | 1.39 | 0.91-2.12 | .129 | 0.94 | 0.66-1.33 | .711 |
| Diagnosis | 1.26 | 0.85-1.88 | .249 | 0.89 | 0.66-1.20 | .440 |
| Language | 0.99 | 0.97-1.01 | .498 | 1.00 | 0.98-1.01 | .708 |
| Age | 0.94 | 0.78-1.12 | .453 | 1.07 | 0.91-1.25 | .416 |
| FSIQ | 1.02 | 1.00-1.05 | .09 | 1.00 | 0.99-1.02 | .656 |
| Attention | 1.00 | 0.94-1.06 | .963 | 0.99 | 0.94-1.05 | .838 |
| Memory | 1.00 | 0.98-1.01 | .871 | 1.00 | 0.98-1.01 | .568 |
