# Supplementary material for: Young Children with ASD Use Lexical and Referential Information During On-line Sentence Processing
Source: Front Psychol. 2016 Feb 19;7:171. doi: 10.3389/fpsyg.2016.00171 (PMC4759258; doi:10.3389/fpsyg.2016.00171)
Supplement: Supplementary file 2 [file Data_Sheet_1.DOCX]

Appendix 1: Stimuli used in Task 1

| Target | Verbs (Expected/Unexpected) | Display |
| --- | --- | --- |
| Water | Drink/Choose | Water, Cheese, Plant, Shoe |
| Football | Kick/Buy | Football, Train, Chocolate, T-shirt |
| Cake | Eat/Move | Cake, Toy, Ball, Cup |
| Book | Read/Open | Book, Bag, Box, Jar |
| Dog | Walk/Feed | Dog, Cat, Rooster, Goldfish |
| Hat | Wear/Take | Hat, Apple, Book, Computer |
| Ladder | Climb/Move | Ladder, Car, Paper, Phone |
| Food | Cook/Buy | Food, Box, Bicycle, Plant |

Appendix 2: Stimuli used in Task 2

| Test Sentences | | Display |
| --- | --- | --- |
| Expected condition | Ambiguous condition |  |
| The man will chop the tree with the axe | The man will chop the tree with the leaves | Tree with no leaves, Tree with leaves, Leaves, Axe |
| The girl will cut the cake with the knife | The girl will cut the cake with the candle | Cake with candle, Cake, Candle, Knife |
| The boy will poke the dog with the stick | The boy will poke the dog with the scarf | Dog with scarf, Dog, Scarf, Stick |
| The lady will break the vase with the hammer | The lady will break the vase with the flowers | Vase, Vase with flowers, Flowers, Hammer |
| The girl will tickle the frog with the feathers | The girl will tickle the frog with the hat | Frog with hat, Frog, Hat, Feather |
| The lady will wipe the girl with the towel | The lady will wipe the girl with the dress | Girl in a dress, Girl in tracksuit, Dress, Towel |
| The girl will wash the plate with the sponge | The girl will wash the plate with the spoon | Plate with spoon, Plate, Spoon, Sponge |
| The man will cover the book with the cloth | The man will cover the book with the ribbon | Book with ribbon, Book, Cloth, Ribbon |
